# Understanding Diabetic Neuropathy: Focus on Oxidative Stress

**DOI:** 10.1155/2020/9524635

**Published:** 2020-07-31

**Authors:** Lei Pang, Xin Lian, Huanqiu Liu, Yuan Zhang, Qian Li, Yin Cai, Haichun Ma, Xin Yu

**Affiliations:** ^1^Department of Anesthesiology, The First Hospital of Jilin University, Jilin, China; ^2^Department of Urology, The First Hospital of Jilin University, Jilin, China; ^3^Department of Ophthalmology, The First Hospital of Jilin University, Jilin, China; ^4^Department of Anaesthesiology, The University of Hong Kong, Hong Kong SAR, China; ^5^Department of Hand Surgery, The First Hospital of Jilin University, Jilin, China

## Abstract

Diabetic neuropathy is one of the clinical syndromes characterized by pain and substantial morbidity primarily due to a lesion of the *somatosensory nervous system*. The burden of diabetic neuropathy is related not only to the complexity of diabetes but also to the poor outcomes and difficult treatment options. There is no specific treatment for diabetic neuropathy other than glycemic control and diligent foot care. Although various metabolic pathways are impaired in diabetic neuropathy, enhanced cellular oxidative stress is proposed as a common initiator. A mechanism-based treatment of diabetic neuropathy is challenging; a better understanding of the pathophysiology of diabetic neuropathy will help to develop strategies for the new and correct diagnostic procedures and personalized interventions. Thus, we review the current knowledge of the pathophysiology in diabetic neuropathy. We focus on discussing how the defects in metabolic and vascular pathways converge to enhance oxidative stress and how they produce the onset and progression of nerve injury present in diabetic neuropathy. We discuss if the mechanisms underlying neuropathy are similarly operated in type I and type II diabetes and the progression of antioxidants in treating diabetic neuropathy.

## 1. Introduction

Diabetic late complications are described as macrovascular complications comprising cardiovascular diseases and microvascular complications, including retinopathy, nephropathy, and neuropathy [1]. Diabetic neuropathy, one of the clinical syndromes, is characterized by pain and substantial morbidity primarily due to a lesion of the *somatosensory nervous system* [2, 3]. The most common clinical pattern is the neuropathy of the feet and the hands with a distal-to-proximal gradient of severity [1–3]. Currently, the only treatment for diabetic neuropathy is glucose control and foot care [2–5].

There are two major predictors of diabetic neuropathy: the duration of diabetes and the levels of haemoglobin A1c [6]. The latter is commonly associated with metabolic factors, genetic risk factors, environmental factors, common cardiovascular risk factors, and poor glycemic control [7–9]. The question, as to by what molecular mechanisms that diabetes mellitus could target sensory neurons, remains unclear [10]. However, a novel concept involving oxidative stress as a potent causative factor of diabetic neuropathy has been put forward [9, 10].

Oxidative stress is caused by an imbalance between production of reactive oxygen species (ROS) and antioxidant systems. It modulates functions of nerve cells through many molecular signalling pathways [10, 11]. Impaired glucose metabolism in diabetes is a critical mechanism to induce oxidative stress as a result of shunting excess glucose to other metabolic or nonmetabolic pathways [7, 8]. This causes accumulation of the toxic metabolites and overconsumption of nicotinic acid adenine dinucleotide phosphate (NADPH). These converge to increase intracellular redox stress and abnormal modifications on protein [12], lipid [13], and DNA [14], thereby adding mitochondrial injury and causing overproduction of ROS. This damages the peripheral nervous system, as indicated by loss of Schwann cells, myelinated axons, and sensory neurons located in the dorsal root ganglia [10, 15, 16]. Meanwhile, insufficient mitochondrial energy production loses the ability to traffic down axons, thereby further promoting axonal injury [17], causing many chronic degenerative diseases, including diabetic neuropathy [7, 8, 18–20].

In this review, we discuss the following points. Firstly, how impaired pathway(s) of glucose metabolism in diabetes lead to oxidative stress, thereby producing the onset and progression of nerve injury present in diabetic neuropathy. The altered pathways include polyol pathway, the hexosamine pathway, the advanced glycation end products (AGE), and protein kinase C (PKC) pathway. Secondly, are the mechanisms underlying neuropathy in type I and type II diabetes distinct? Thirdly, is any antioxidative drug specific and effective for relieving painful diabetic neuropathy? Finally, to highlight areas of research needed for improving the fate of patients with painful diabetic neuropathy.

## 2. General Concept of Oxidative Stress

**Figure 1:**
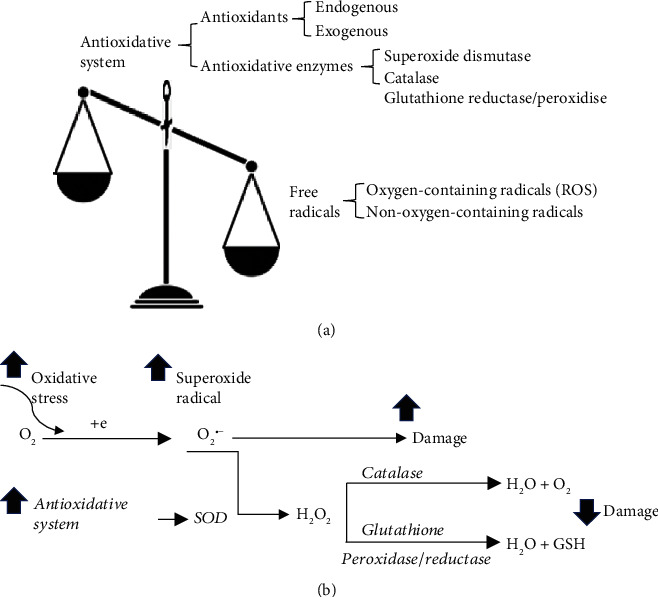
Components of oxidative stress and working mechanism of antioxidative system. (a) Oxidative stress consists of two components as the free radicals and the antioxidative system. The term of oxidative stress describes a condition when the generation of free radicals and the antioxidative system is imbalanced. Free radicals can be the radicals with or without reactivity of oxygen. Antioxidative system consist of antioxidants, that are derived either endogenous or exogenous, and antioxidative enzymes, such as superoxide dismutase, glutathione reductase/peroxidise, and catalase. (b) Antioxidative system works through mechanisms suppressing and scavenging not only free radicals but also *de novo* antioxidant. Superoxide (O_2_^•−^) is the major ROS produced in the mitochondria with increased oxidative stress. However, conversion of O_2_^•−^ to H_2_O by coupled reactions of superoxide dismutase (SOD), catalase, and glutathione reductase/peroxidise is accompanied by the formation of GSH and elimination of the detrimental effect of O_2_^•−^ on damage the cells.

The term of oxidative stress describes a condition (Figure 1(a)) when the balance between the generation of free radical and antioxidant system is unfavorable [21, 22]. A free radical can be defined as any molecular species capable of either donating or accepting an electron therefore behaving as oxidants [22], while antioxidants are the molecules stable enough to neutralize the free radicals, thereby maintaining a balance. Despite the chemical structural differences, free radicals share similar mechanisms for damage at the level of biomolecules [23].

Among the free radicals, ROS are the oxygen-containing free radicals as the natural by-products of the metabolism of oxygen, including hydroxyl radical, superoxide anion radical, hydrogen peroxide, oxygen singlet, hypochlorite, nitric oxide radical, and peroxynitrite radical. They are derived within organelles including peroxisomes, endoplasmic reticulum, and mitochondria, the major site of ROS production.

Antioxidant system consists of antioxidants and antioxidative enzymes (Figure 1(a)). The resources of antioxidants can be endogenous and exogenous. Glutathione is the most abundant endogenous antioxidant in most cell types with the reduced form (GSH) as biologically active [24]. The exogenous antioxidants are derived from either diet or supplement, such as vitamin A, C, and E [21, 25] and antioxidant minerals (copper, zinc, manganese, selenium). The major antioxidative enzymes, including superoxide dismutase, glutathione reductase/peroxidise, and catalase, metabolize free radicals to nontoxic intermediates. They work in synergy (Figure 1(b)) through mechanisms not only suppressing and scavenging free radicals before they can damage cells, but also repairing *de novo* antioxidants [24, 26].

## 3. Diabetic Neuropathy

### 3.1. General Aspects

Diabetic neuropathy, a damage occurred in sensory neurons, causes neuropathic pain with either central or peripheral syndromes in different patterns (e.g., pain and numbness) [7, 9]. Clinically, the most common pattern is the distal symmetric polyneuropathy of the feet and hands, with a distal-to-proximal gradient of severity [1, 9, 10, 27]. Of note, pain is reported by approximately one third of patients with diabetes, regardless of associated neurological deficits [27].

The cause of diabetic neuropathy has been attributed to diabetes [8, 9], which is a metabolic disorder characterized by impaired glucose metabolism with chronic hyperglycaemia and dysfunction of endogenous insulin (insufficiency of secretion as type I or action as type II). Based on the large clinical studies in patients with type I or type II diabetes, a strong correlation between chronic hyperglycaemia and diabetic microvascular complications has been established [28–33]. At least 50% of individuals with diabetes develop diabetic neuropathy with time [34].

Increased glucose levels in diabetes affect primarily those cells that have a limited capacity to regulate their glucose intake, including vascular cells, Schwann cells, and neurons of the peripheral and central nervous systems [35, 36]. It is unclear whether high glucose triggers axonal degeneration by promoting intrinsic programmes within axons [36–38], nor is whether peripheral axons or their associated Schwann cells, the first target. Clinical findings have demonstrated that Schwann cells are targeted in patients with diabetic neuropathy [39]. Experimental studies in diabetic rodents have associated endoplasmic reticulum stress with diabetes-mediated peripheral nerve damage [40]. At the early stage of diabetes, hyperglycaemia causes abnormalities in blood flow and in vascular permeability [7]. With time, impaired glucose metabolism reduces intracellular levels of NADPH and decreases the synthesis of myo-inositol that is particularly required for the normal function of nerves [41, 42]. Collectively, diabetic neuropathy might be caused by a direct effect of hyperglycaemia on damaging cells [43, 44] and an indirect effect on affecting cellular functions [7, 45]. Loss of microvascular cell occurs, in parallel with reduced production of endothelial and neuronal cells, which leads to degeneration of peripheral nerves [46].

### 3.2. Diabetic Neuropathy in Type I and Type II Diabetes

The major predictors of diabetic neuropathy are the duration of diabetes and the blood levels of haemoglobin A1c (HbA1c) [6]. The severity of diabetic complications correlates with the severity of hyperglycaemia, suggesting that the complications are triggered by the elevation in glucose levels [28]. However, rapid glucose control significantly increased the risk of severe hypoglycemic episodes [47] and resulted in treatment-induced neuropathy in both type I and type II diabetes [48–50].

Diabetic neuropathy can be found late in type I diabetes but early in type II diabetes, and the cause of this occurrence remains unclear. As the consistent feature between type I and type II is hyperglycaemia, one would assume that controlling hyperglycaemia would be the best preventive treatment for diabetic neuropathy regardless the diabetic type. Of interest, the incidence of neuropathy is higher in diabetic patients with type II than those with type I [51–54], whereas the prevalence of diabetic neuropathy was similar in type II diabetic patients [55–57] as seen in type I [6, 58]. Clinically, efficient glucose control significantly reduced or delayed the incidence of developing neuropathy in type I diabetic patients [59, 60], whereas it remains elusive with type II diabetes [29, 47, 61–63]. In addition, lowing haemoglobin A1c in type II diabetic patients has little effect on diabetic neuropathy [64], whereas a greater improvement has been observed in type I diabetic patients after 18 months of glycemic control [48]. Collectively, it suggests that hyperglycaemia is not the prime driving cause of all complications [35, 65], and mechanisms underlying diabetic neuropathy in type I and type II diabetes could be fundamentally different [47].

The most recent findings with alterations in DNA methylation have been suggested as a contributor to diabetic neuropathy in type I and type II diabetic patients [14, 66]. Of interest, spliceosome dysregulation has been proposed as a key neurodegenerative mechanism underlying diabetic neuropathy in type I diabetic patients [67]. Spliceosome is a complex assembled from small nuclear RNA and proteins in nucleus and required to catalyze pre-mRNA splicing in nuclear speckles [68]. Whether splicing abnormalities are identifiable in type II diabetes remains unexplored.

The current approaches to managing diabetic neuropathy focus on improving glycaemic control, mainly in type I diabetic patients, and lifestyle modifications, mainly in type II diabetic patients [35, 69]. Although diabetic neuropathy is the strongest predictor of mortality in type II diabetes, it remains the only microvascular complication of diabetes without a specific treatment owing to our lack of basic understanding of this disease.

## 4. Oxidative Stress in Diabetic Neuropathy

### 4.1. Main Sources of ROS Production in Diabetic Neuropathy

The main proof of oxidative stress involvement in diabetic neuropathy was the accumulation of free radicals and reduced activity of antioxidant enzymes in the diabetic animals with diabetic neuropathy, and the effect was ameliorated, in parallel with the alleviation of symptoms, upon antioxidant treatment [70]. Ample evidence strongly support that hyperglycaemia leads to increased oxidative stress that plays a pivotal role in the development of diabetic neuropathy [7] by damaging the cells including endothelial, retinal, mesangial, and neural cells [2, 8].

**Figure 2:**
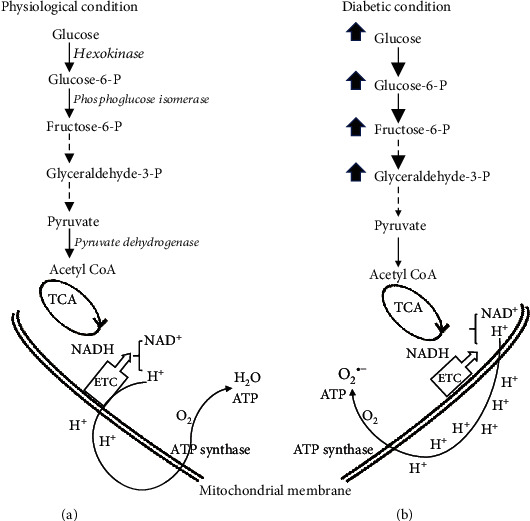
Major glucose metabolism pathway under physiological condition and diabetic condition. (a) Under a physiological condition, intracellular glucose is converted to glucose-6-phosphate, via hexokinase, then following isomerization to fructose-6-phosphate. Along glycolysis pathway, glyceraldehyde-3-phosphate travels down to pyruvate and acetyl CoA via pyruvate dehydrogenase, which then enters tricarboxylic acid (TCA) cycle. NADH, as electron carrier and generated during the process of glycolysis and glucose oxidation, can donate reducing equivalents to the mitochondrial electron transport chain (ETC), thereby creating a proton gradient that is used by ATP synthase to produce ATP and converting O_2_ to H_2_O. (b) Under diabetic condition, glucose metabolism is impaired, causing accumulation of glucose and the glycolytic intermediates, resulting mitochondrial injury, thereby converting O_2_ to superoxide radical (O_2_^•−^) instead of H_2_O. As a result, ATP production is reduced.

Impaired mitochondrial glucose oxidation is believed as a main source of ROS production in diabetes [7, 8]. To understand how hyperglycaemia increases ROS production, a brief overview of glucose metabolism is helpful (Figure 2(a)). Under a physiological condition, after being taken-up, intracellular glucose is converted to glucose-6-phosphate, then followed by glycolysis and oxidation to produce NADH and acetyl CoA. Electrons carried by NADH are transferred to oxygen following mitochondrial electron transport chain (ETC), along with pumping protons out of the mitochondrial matrix thereby creating a proton gradient that is used by ATP synthase to produce ATP (Figure 2(a)). Under normal condition, there are only 0.2-2% of the electrons in the ETC leaking out to produce ROS [71], and neurons have sufficient capacity to remove ROS by innate cellular antioxidants (Figure 1(b)) [72]. Under a diabetic condition, however, impaired glucose metabolism shunts glucose or intermediates of glycolysis to other metabolic and nonmetabolic pathways (Figure 2(b)), which causes mitochondrial injury with higher rates of protons returning to the mitochondria without generating ATP and an overwhelming production of ROS in a neuron [11]. Axons are rich of mitochondria, having a direct access to nerve blood supply. The inability of the neuron to detoxify the excess ROS together with insufficient ATP production leave axons being more susceptible to ROS-mediated damage in hyperglycaemia [11], in part because of their dependence on local mitochondria for energy, which in turn precipitates axonal degeneration [11].

### 4.2. Molecular Mechanisms of ROS Production in Diabetic Neuropathy

**Figure 3:**
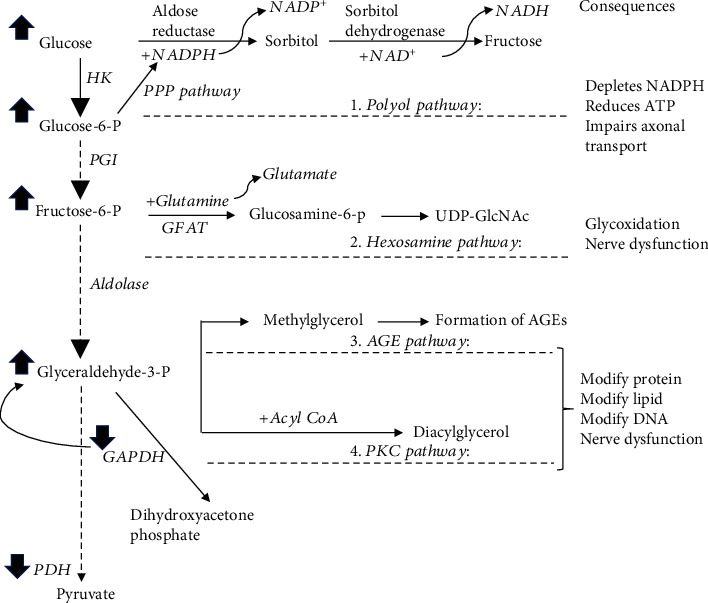
Four damaging pathways that can explain the detrimental effects of ROS in hyperglycaemia-induced diabetic neuropathy. The impaired glucose metabolism in diabetic condition causes an accumulation of glucose and glycolytic intermediates, which, instead of travel along glycolysis pathway, shunts to other metabolic or nonmetabolic pathways, resulting activation of the polyol pathway, hexosamine pathway, and AGE and PKC pathway. Superoxide inhibits glyceraldehyde-3-phosphate dehydrogenase (GAPDH) activity, which is proposed to be a reason causing accumulation of all the glycolytic intermediates. Pentose phosphate pathway (PPP pathway) is to generate NADPH, which is used in polyol pathway. GFAT: glutamine-fructose-6-phosphate aminotransferase; GAPDH: glyceraldehyde-3 phosphate dehydrogenase; PDH: pyruvate dehydrogenase.

There are four damaging pathways (Figure 3) that can explain the detrimental effects of ROS in hyperglycaemia-induced diabetic neuropathy, including polyol pathway and hexosamine pathway that have been consistently observed in patients with diabetic neuropathy [73–76]. The AGE and PKC pathway modify proteins, lipids, and DNAs via a direct [77–80] or an indirect effect of glucose [81, 82]. All of which are linked to diabetic neuropathy by a single event: overproduction of ROS [7], which is a consistent differentiating feature common to all cell types that are damaged by hyperglycaemia [8].

#### 4.2.1. Activated Polyol Pathway

The polyol pathway is a two-step metabolic process (Figure 3), promoted by a mass action of excess glucose to activate aldose reductase. Aldose reductase, a cytosolic protein, normally has low affinity to glucose, and function for reducing toxic aldehydes in tissues such as nerve, retina, lens, glomerulus, and vascular cells [83, 84]. In many of which, glucose moves freely across the cell membrane independent of insulin, and intracellular levels of glucose rise with hyperglycaemia in parallel with an increased affinity of aldose reductase for glucose. This favors the excess glucose to generate sorbitol, instead of down to glycolysis, with consumption of nicotinamide adenine dinucleotide phosphate (NADPH) to NADP^+^ (Figure 3). Sorbitol is subsequently oxidized to fructose through sorbitol dehydrogenase, with NAD^+^ as a cofactor. To note, compared to glucose, glyceraldehyde 3-phosphate, a glycolytic intermediate, has been suggested as a relevant substrate for aldose reductase, owing to its higher affinity to aldose reductase under pathological conditions [85].

The well accepted mechanism for hyperglycaemia-induced polyol pathway is the increased redox stress due to the consumption of NADPH, which is derived from pentose phosphate pathway for generating GSH from glutathione (Figure 3). This notion is further supported by the observation that overexpression of human aldose reductase in diabetic mice reduced the expression of regulatory genes for regenerating glutathione [86]. Meanwhile, excess fructose, as a product, promotes glycation and further depletion of NADPH thereby causing and exacerbating intracellular oxidative stress.

Relevant to diabetic neuropathy, accumulation of sorbitol and fructose were observed in the peripheral nerves of diabetic rats [87], while shunting glycolytic intermediates to polyol pathway also promotes glycation and formation of diacylglycerol in dorsal root ganglia of the diabetic mice [88]. Inhibiting aldose reductase prevented accumulation of sorbitol and fructose in peripheral nerves of the diabetic rats [89, 90] and restored diabetes-induced defect in nerve conduction velocity in diabetic dogs [91]. In addition, patients with a high aldose reductase expression are commonly having an early diabetic neuropathy relative to the patients with a low aldose reductase expression [92, 93]. Thus, inhibiting the polyol pathway continues to be a drug target in the treatment of diabetic neuropathy.

#### 4.2.2. Activated Hexosamine Pathway

The well accepted mechanism that hexosamine pathway contributes to diabetic neuropathy is the effect of intracellular UDP-GlcNAc on modification of proteins [94, 95]. Under physiological condition, hexosamine pathway is a minor branch of the glycolytic pathway [96, 97] with only 2-5% [97] of fructose-6-phosphate being converted to glucosamine-6-phosphate by glutamine-fructose-6-phosphate aminotransferase (GFAT), the rate limiting enzyme. Under hyperglycaemia condition, however, the increased production of mitochondrial ROS inhibits glyceraldehyde-3 phosphate dehydrogenase activity, a glycolytic enzyme (Figure 3), thereby blocking fructose-6-phosphate flow through glycolysis [98]. Subsequently, glucosamine-6-phosphate along with acetyl-CoA and uridine-5′-triphosphate are used to produce the amino sugar uridine-5′-diphosphat-N-acetylglucosamine (UDP-GlcNAc) [97], which controls activity of O-linked N-acetylglucosamine transferase [99]. The latter is a cytosolic and nuclear enzyme, catalyzing a reversible posttranslational protein modification by transferring GlcNAc to specific serine and threonine residues on proteins [99, 100]. Of particular interest at proteins modified by O-GlcNAcylation are insulin receptor substrates-1 and 2 [101, 102] as well as glucose transporter 4 [103].

Relevant to diabetic neuropathy, an increase in GFAT activity and UDP-GlcNAc concentrations was evident in muscle of ob/ob mice [104]. Conversely, a reduced UDP-GlcNAc concentration was observed, in parallel with improved insulin sensitivity in muscles of the rats with chronic caloric restriction [104]. Clinically, GFAT activity is increased in muscle biopsies obtained from insulin resistant patients with type II diabetes [105], while insulin resistance is improved markedly by insulin treatment in patients with severely insulin resistant, uncontrolled, obese, type II diabetes, concomitant with 40% increase in the levels of UDP-GlcNAc in muscle [106]. In contrary, a positive correlation among UDP-GlcNAc circulating levels of FFA and leptin was found in adipocytes, but not in muscle of type II diabetic patients relative to nondiabetic control [107]. Hyperglycaemia-induced overmodification of proteins by glucosamine results in pathological changes in gene expression, especially transcription factors, which contribute to the pathogenesis of diabetic complications with the strongest evidence for the role in diabetic complications [102, 108]. However, it is not yet clear what kind of peripheral nerve proteins can be modified by activated hexosamine pathway following diabetes, nor is the causal connection. Thus, the contribution of hexosamine pathway to diabetic neuropathy remains to be further explored.

#### 4.2.3. Activated AGE and PKC Pathway

The common feature of both pathways is the modification of proteins thereby modulating diabetic complications via activating transcription factors. Activation of hexosamine pathway causes an elevated level of glyceraldehyde-3 phosphate, which upon conjugation with fatty acid, produces diacylglycerol [109, 110] to activate PKC. Thus, both activated polyol pathway and hexosamine pathway in diabetes can activate AGE and PKC pathway.

*(1) Activation of AGE Pathway.* AGEs are intracellular and extracellular adducts formed by covalent linking reducing sugars or its metabolites to lysine or arginine on proteins [111–113]. Hyperglycaemia is recognized as the primary initiating event in the formation of AGEs [78]. There are two forms of AGEs precursors, glyoxal and methylglyoxal, which can be generated through three major pathways: auto-oxidation of glucose to form glyoxal, a smallest dialdehyde [80]; abnormal metabolism of glyceraldehyde-3 phosphate from glycolysis to form methylglyoxal; and degradation of glyceraldehyde and glycolaldehyde (fructose-lysine adducts) to form both glyoxal and methylglyoxal. Compared to glyoxal, methylglyoxal is highly reactive and causing vascular endothelial cells to be more sensitive to damage [114].

Relevant to diabetic neuropathy, the well accepted mechanism is that extracellular AGEs interact with a specific AGE receptor: known as RAGE on the cell surface [115], causing overproduction of ROS, thereby activating nuclear factor kappa B (NF-*κ*B) to initiate multiple pathological changes in gene expression [116]. Activation of AGE-RAGE-NF-*κ*B axis appears to be sustained in both clinical [117] and laboratory settings [112, 117]. In the streptozotocin-induced diabetic mice, knockdown of RAGE gene significantly improved electrophysiological and anatomical markers of diabetic neuropathy [112] as well as restored pain perception in sciatic nerves [117], in parallel with a decreased expression of NF-*κ*B in peripheral nerves [117], and particularly in Schwann cells [112]. Clinically, activated NF-*κ*B was colocalized with RAGE within the vasa nervorum in the sural nerve biopsies from the patients with diabetic neuropathy [117]. In relevant, diabetic patients have increased expression of endothelia RAGE and less collateral vessels, compared with nondiabetic controls, which contributes to increased rates of lower limb amputation [118]. Thus, AGE-RAGE-NF-*κ*B axis might promote diabetic neuropathy through its impact on microvessels within the sensory neurons. Up to date, effective treatment modalities of AGE-induced nerve injury is not available clinically [67].

*(2) Activation of Protein Kinase C.* Activation of classic PKC is dependent on both Ca^2+^ ions and phosphatidylserine and is greatly enhanced by diacylglycerol [119]. The primary function of PKC is phosphorylating targeted proteins, which in turn operating on gene expression in diabetes, thereby mediating abnormalities of blood flow and permeability via inhibiting NO production [120] or activating NF-*κ*B [121] and microvascular matrix proteins [122] in both diabetic patients [123] and animal models [124].

Of relevance, the PKC *β*-isoform in particular has been linked to the development of diabetic nephropathy in the diabetic animal models, such as in the streptozotocin-induced diabetic rats; PKC*β* inhibitors actually improve motor nerve conduction velocity and endoneurial blood flow [125, 126]. However, clinical studies using ruboxistaurin, a selective PKC*β* inhibitor, for treatment of painful diabetic neuropathy did not achieve significance [127]. Based on six randomized controlled trials, it was concluded that PKC*β* inhibitor offered no benefit in the treatment of diabetic neuropathy [128]. Up to date, the role for PKC activation in diabetic neuropathy remains unclear.

## 5. Management of Treating Diabetic Neuropathy in Relation to Oxidative Stress

### 5.1. Assessment

There are two newer techniques, besides a number of other tools [65], for clinician to assess diabetic neuropathy, including the visual quantification of intraepidermal nerve fibers through skin biopsy for peripheral vs. MR imaging for central neuropathy, which allows noninvasive *in vivo* imaging of corneal nerves [129, 130].

### 5.2. Newest Approach in Pain Medicine

The newest approach was released on May 2020 by Vienna University of Technology that chronic pain can be reduced by stimulating the vagus nerve in the ear with a tiny electrodes, in which a 3D computer model is created to calculate the optimal stimulation of nerve branches [131, 132]. The approach has now been successfully tested on patients [132]. Although this is not specifically for treatment of diabetic neuropathy, it is an important step forward.

In clinical practice, the assessment with combination pharmacotherapy is often applied for managing diabetic neuropathy. Of interest, pharmacological approaches aiming at targeting antioxidative stress are the few strategies that reduce pain in diabetic neuropathy patients in clinical trials [133–138].

### 5.3. Targeting Oxidative Stress

**Figure 4:**
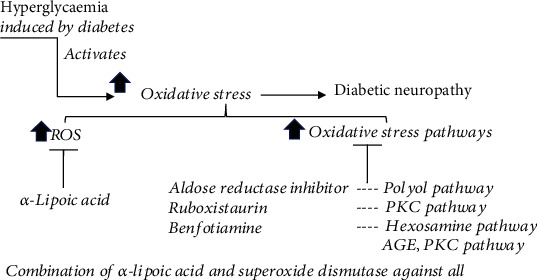
Strategies of antioxidative drugs in treating diabetic neuropathy include directly against ROS and against individual oxidative stress pathways. The listed drugs showed effect on improving symptoms of diabetic neuropathy. However, none of them have been FDA-approved due to the lack of clinical significance.

Several strategies aiming at antioxidative stress have been employed (Figure 4) to combat nerve dysfunction in diabetes, including directly against ROS, against individual oxidative stress pathways, or targeted at mitochondria.

### 5.4. Targeting ROS

Including *α*-lipoic acid (ALA), vitamins A, C, and E, acetyl L-carnitine, taurine, and melatonin, taurine, acetyl L-carnitine, and N-acetylcysteine have been demonstrated to reduce the progression of diabetic neuropathy [139], whereas the effect of vitamins A, C, and E in diabetic neuropathy needs more research to ascertain [9, 21]. ALA is thought to be a valuable therapeutic option for diabetic neuropathy as the treatment ameliorated the symptoms of diabetic neuropathy in the clinical trials [135, 137, 138]. ALA is a water- and fat-soluble compound known to reduce oxidative stress by inhibiting hexosamine and AGEs pathways [140]. The combination of ALA and superoxide dismutase has improved symptoms and electroneurographic parameters in the patients with diabetic neuropathy [141]. Currently, ALA has been licensed in Germany to treat symptomatic diabetic neuropathy with 600 mg daily dosage [142].

### 5.5. Targeting Individual Oxidative Stress Pathways

Including aldose reductase inhibitors, anti-AGE agents, and PKC inhibitors, in contrast to ruboxistaurin, a specific PKC*β* inhibitor failed to achieve its clinical significance; aldose reductase inhibitors and anti-AGE agents have shown the therapeutic effect.

Aldose reductase inhibitors are used to reduce the flux of glucose into the polyol pathway. The positive effect of inhibiting aldose reductase on diabetic neuropathy includes enhancing sural motor and sensory nerve conduction velocities, improving wrist and ankle *F*-wave latency together with and alleviating neuropathic pain [143], which have been observed in diabetic mice [143] and in diabetic patients [144]. However, its efficacy needs further investigation [145].

Anti-AGE agents are used to prevent the formation and accumulation of AGEs. Benfotiamine, a lipid-soluble analogue of vitamin B_1_, has the effect on preventing the activation of the hexosamine pathway and the AGE and PKC pathway induced by diabetes [7, 8]. Importantly, benfotiamine has shown the therapeutic efficacy in the patients with diabetic neuropathy [134, 146, 147] and in diabetic rats [8, 133], which suggests that benfotiamine may extend the treatment option for diabetic neuropathy based on causal influence on impaired glucose metabolism [134].

The clinical translation is challenging, as none of these drugs are currently FDA-approved yet, in part, due to the failure in clinical trials [148, 149]. One of the common contributors to the failure in the clinical trials is the irreproducibility of the improvement in the treated animal models vs. in humans, for example, sorbinil (an aldose reductase inhibitor), which was shown to inhibit diabetes-induced nerve conduction deficit in streptozotocin-induced diabetic rats [150]; however, it did not produce similar results in humans [151]. Another concern is the side effect, such as photosensitive skin rash, discouraging the further usage [152]. Extensive preclinical research is still on going to investigate further mechanisms and new targets with improved efficacy and safety for treating diabetic neuropathy.

## 6. Conclusion

Although considerable research has been devoted to understanding mechanisms of diabetic neuropathy in general, treatment options to eliminate the initial causes are still “lacking. There have been disparities between the results obtained from animals and human studies. Currently, genetic- or diet-induced animal models are commonly used to investigate neuropathy in type II diabetes, while high-dose streptozotocin-induced diabetic animal models are used to mimic the metabolic phenotype of type I diabetes. However, no single rodent model accurately mimics human diabetic neuropathy [153] with the major problems not only in pain assessment that is hard to measure in rodents [154] but also in the knowledge about the molecular mechanisms. Most knowledge about mechanisms of diabetic neuropathy was gained in genetically homogenous male rodents, while patients vary in sex, ethnicity and genetic background, age, and duration of diabetes. Thus, these factors should be considered in designing the individualized treatment plans for patients with diabetic neuropathy.

**Figure 5:**
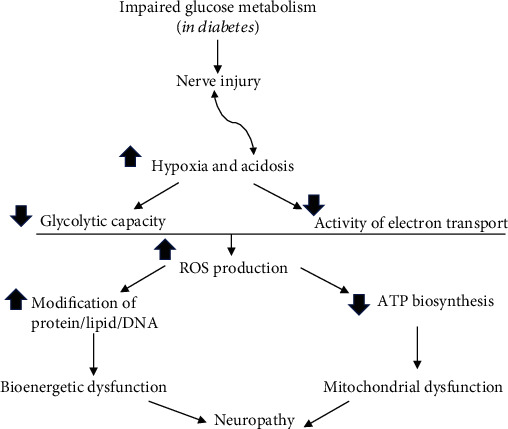
Summary of the major contributors to diabetic neuropathy. Diabetes-induced impairment of glucose metabolism causes hypoxia and acidosis, which contributes to and exacerbates the nerve injury. As a result, both glycolytic capacity and activity of electron transport chain are reduced, leading to overproduction of ROS, which, not only reducing ATP production but also initiating various modifications on protein/lipid and DNA. Thus, mitochondrial and bioenergetic dysfunction leads to neuropathy.

Hyperglycaemia-induced oxidative stress remains the most accepted mechanism for the progression of diabetes to diabetic neuropathy (Figure 5). The impaired glucose metabolism in diabetes leads to hypoxia and acidosis, which trigger other abnormalities responsible for mitochondrial and bioenergetic dysfunction by increasing ROS production to cause membrane hyperexcitability and reduction of ATP production. Treatment options with antioxidants have been investigated; none are satisfactory. Treatment which repairs nerves, in patients with diabetic neuropathy, has yet to be translated into clinical trials. Thus, future research must establish the most efficacious drug combinations on combating hyperglycaemia and oxidative stress for the prevention/treatment of diabetic neuropathy, in addition to explore the new mechanisms. Clinically, predictors and biomarkers need to be validated for both clinical trials and clinical practice.
